# Emergency medical team reclassification in WHO’s Western Pacific Region: continuous learning and improvement of health emergency response capacities

**DOI:** 10.5365/wpsar.2025.16.3.1263

**Published:** 2025-09-01

**Authors:** Sean T Casey, Roy Cosico, Jorge Salamanca, Camila Lajolo, Erin Noste, Kathleen Warren, Chandra Gilmore, Eystein Grusd, Jan-Erik Larsen, Pierre-Yves Beauchemin, Flavio Salio

**Affiliations:** a World Health Organization Regional Office for the Western Pacific, Manila, Philippines.; b School of Population Health, Faculty of Medicine and Health, University of New South Wales, Sydney, New South Wales, Australia.; c World Health Organization, Geneva, Switzerland.; d Department of Emergency Medicine, University of California San Diego, San Diego, California, United States of America.; e Oslo Metropolitan University, Oslo, Norway.

Emergency medical teams (EMTs) are groups of self-sufficient and equipped health professionals who deploy in a structured and coordinated manner in response to emergencies with health consequences. EMTs may be hosted by governments, nongovernmental organizations, private companies, academia, militaries, or through the International Red Cross and Red Crescent Movement. (*1*-*3*)

Since 2013, with the initial publication of the *Classification and minimum standards for foreign medical teams in sudden onset disasters* and the subsequent 2021 publication of the updated *Classification and minimum standards for emergency medical teams* (both known as the EMT Blue Book), the World Health Organization (WHO) has worked with Member States, organizations and partners to establish and apply common principles and core standards for medical teams around the world engaging in health emergency response. (*1*, *2*) The recently published *Emergency Medical Teams 2030 Strategy* set ambitious targets, including enhancing quality-assurance mechanisms for both national and international EMTs. (*4*)

EMTs pursue quality assurance through a structured mentoring and peer review process, culminating in verification and classification. (*2*) As of 31 May 2025, 55 EMTs from all six WHO regions had successfully achieved classification, including 17 EMTs from WHO’s Western Pacific Region, with more than 15 EMTs from the Region pursuing classification. (*3*, *5*, *6*) EMT classification is valid for 5 years, after which a team must undergo reclassification. (*2*)

In 2022, the EMT Strategic Advisory Group (SAG) endorsed *The reclassification cycle for emergency medical teams*, a guidance document for reclassification of EMTs (unpublished), which was developed by a global EMT Reclassification Technical Working Group. Designed to be less intensive than the initial classification process, EMT reclassification involves a team confirming its continued interest in classification through submission of a “re-engagement” form and an “improvement dossier” detailing the EMT’s deployment history and improvements made since initial classification. This process ensures the quality of health services during emergencies and actions taken to meet the standards and recommendations introduced in the 2021 EMT Blue Book. (*2*, *5*) A key element of the quality assurance is peer feedback provided by the verification team to the EMT during the initial classification visit, which is reviewed as part of reclassification. EMTs pursuing reclassification may request a mentor to be appointed from a WHO-classified EMT to guide them through the process. Allocated mentors may be a designated and trained EMT mentor or may be a senior member of a WHO-classified EMT with demonstrable experience with the classification/reclassification process.

Following the submission of the improvement dossier, EMTs undergo an online desk review to present their continued compliance and improvements made following classification. The external verification team is composed of WHO headquarters and/or regional EMT focal points, as well as external peer reviewers from classified EMTs. Following the online desk review, a 1-day in-person site visit is conducted at a team’s physical EMT base/camp to inspect the EMT’s continued compliance with published minimum standards, lessons learned through their work in the 5 years following classification, and improvements made (**Table 1**). At the end of the reclassification visit, the panel may conclude that an EMT should be reclassified for an additional 5 years or recommend a re-evaluation 6 months later if deficiencies are identified. A site visit may also take place during an EMT deployment as part of an emergency response. In this scenario, the EMT must be deployed in the full capacity for which it was classified (such as an EMT Type 2 deployment for a Type 2 reclassification). This option does not alter the reclassification requirements but instead rearranges the steps, allowing the EMT to undergo quality assurance while deployed, followed by a desk review later.

**Table 1:** EMT capabilities reviewed through the reclassification process

Operational area	Specific area
**EMT capacity and capability**	**Rapidly deployable temporary shelter, outpatient clinic and inpatient facility**
	**Recognized triage system for emergency and surgical care, including acute medical and obstetric conditions**
	**Basic/advanced life support**
	**Capable of a safe uncomplicated delivery with midwifery-level care**
	**Emergency caesarean section and surgical carea**
	**Patient registration and unique patient-identification system in place**
	**Temporary isolation capability**
	**Privacy and confidentiality maintained within the facility**
	**EMT fully staffed with the right technical skill sets and staffing ratios for** **the type of EMT**
**Operating theatre^a^**	**Surgical documentation available and in use**
	**Lighting system sufficient to visualize deep intra-abdominal area**
	**Backup power supply**
	**Cold chain and drug control including locked drug storage**
	**Adult and paediatric anaesthesia care**
	**Emergency surgical (including obstetrics and gynaecological) care**
	**Reconstructive and specialist surgical care**
	**Appropriate climate and vector control measures within the operating theatre area**
	**One-way movement system of surgical instruments and medical devices from contaminated to clean areas for sterilization (i.e. operating theatre to sterilization area)**
	**Standard operating procedure and available equipment for the reversal of sedation**
**Technical services**	**Sterilization**
	**X-ray (±) ultrasounda**
	**Capable of point-of-care and basic rapid detection tests**
	**ABO and Rh screeninga**
	**Walking blood bank (or equivalent) compliant with WHO guidelines for the selection, screening and administration of donor blooda**
	**Documented record of surgical sterilization and traceability proceduresa**
	**Appropriate radiation control measures in place to mitigate time/distance and shielding (i.e. provision of appropriate lead shielding and personal dosimeters; a cordoned-off safety area surrounding the X-ray tent, adequate signage, etc.)a**
	**X-ray procedures in compliance with standards of justified practice (i.e. clear record of requests, rationale and reporting in the patients notes)a**
	**Appropriate laboratory equipment and consumables available, with quality assurance to undertake a walking blood bank or alternativea**
	**Appropriate blood donor screening, testing, donation and administration procedures (i.e. clear record of processes noted in patient and donor notes) in placea**
	**Standard operating procedure in place in case of blood transfusion reactionsa**
**Pharmacy**	**Stock within expiry date, medications are labelled (in local language, where possible) and individually dispensed with authorized prescription**
	**Cold chain/equipment compliance**
	**WHO essential medication list or equivalent (national)**
	**A register for scheduled/controlled substances and dispensing is maintained**
	**Pharmacy stock control system in place**
	**Daily record (ideally twice a day) of external and internal temperature of the pharmacy**
	**Reporting procedure in place to manage medication errors**
**Referral capacity**	**Ability to identify and manage referrals to higher/lower levels of care**
	**Methods of transfer/transport are identified for referral cases**
	**Available equipment and consumables to support patient transfers**
	**Referral form available and in use**
	**Review completeness and accuracy of referral forms (reviewers should indicate the specific forms and the number reviewed)**
**Medical records and reporting**	**System to maintain confidential, individual patient records/reports on a regular basis**
	**Regular reporting using identified national or international reporting format (i.e. Minimum Data Set/MDS)**
	**There is a unique system to identify and verify patient’s identity**
	**EMT has a paper-based, or paper and electronic, or just electronic-based medical records system in place, with a backup mechanism, if required**
	**Appropriate data and patient records management, i.e. safe and secure storage of patient records**
	**Indicate which/how many documents were reviewed**
**Waste management**	**Waste management system to ensure patient, staff and community safety, including segregation, handling, treatment and disposal**
	**Safe handling and disposal of sharps**
	**Waste management area demarcated/fenced**
	**EMT uses a segregated coloured-coding system for waste management**
	**EMT staff use personal protective equipment in handling waste**
	**Note what waste treatment and disposal system is used**
	**Note the measures the EMT takes when a needle-stick/sharps injury is reported**
	**Note use of an incinerator and specify type**
**Water, hygiene and sanitation**	**Adequate quantities of safe drinking-water available**
	**Adequate quantity and quality of water to cover handwashing, personal hygiene, cleaning and laundry needs**
	**Adequate number of toilets and showers for patients and staff (minimum 2 per 100 outpatients and 2 per 20 inpatients)**
	**Handwashing stations available in all key areas of the facility**
	**Number of litres of water/day**
	**Total water storage capacity**
	**Water treatment plan (filtering, sedimentation, chlorination, flocculation and/or other)**
	**Proof of water testing and treatment regimens undertaken**
	**Number of toilets per patient**
	**Disabled access to toilet facilities**
	**Number of toilets/staff**
**Self-sufficiency**	**Shelters: adequate number of shelters available to provide safe and secure working and living conditions for team members and patients**
	**Power: capacity to provide power to all required sources, and suitable lighting sources available for a safe working environment**
	**Communication: emergency communication available, such as BGAN, high-frequency radio, satellite phone, mobile phone**
	**Climate appropriate shelter system is used and able to maintain comfortable internal operating temperatures**
	**Fuel is stored safely and has a supporting hazard management plan**
	**Note the fleet and management system in place**

ABO: A positive and negative blood types, B positive and negative blood types, AB positive and negative blood types, O blood type; BGAN: Broadband Global Area Network; EMT: emergency medical team; Rh: Rhesus positive and negative blood types; WHO: World Health Organization.

^a^ Only applies to Type 2 or Type 3 EMTs, although, as per the 2021 Blue Book, ultrasound may be used to aid in diagnosis where appropriately trained/skilled clinicians are available.

## ACTIONS

By the end of 2024, 10 of the 16 classified EMTs from the Region were due for reclassification. During the COVID-19 pandemic, the EMT SAG granted an extraordinary 2-year extension to mitigate the disruptions caused by the pandemic, ensuring that EMTs remained operational and recognized within the global classification framework.

Western Pacific Region EMTs due for reclassification by the end of 2024 included:

Japan Disaster Relief (JDR) EMT following classification in 2016;
five China International EMTs following classification in 2016 (1), 2017 (1), 2018 (1) and 2019 (2);
Australian Medical Assistance Team (AUSMAT) following classification in 2016;
New Zealand Medical Assistance Team (NZMAT) following classification in 2017;
Aspen Medical following classification in 2018; and
Fiji Emergency Medical Assistance Team (FEMAT) following classification in 2019.

As of 31 May 2025, four of these EMTs – AUSMAT, FEMAT, JDR and NZMAT – had completed the above process. An online desk review was held for the China International EMT (Shanghai) in July 2024, and the in-person visit for this reclassification is expected in 2025. The desk review for the China International EMT (Sichuan) is tentatively scheduled for the second half of 2025. The China International EMT (Macao) is currently preparing for reclassification in 2026. Other EMTs in the Region have initiated the reclassification process.

All four of the reclassified EMTs have deployed since their initial classifications. (*5*) The reclassification visits for AUSMAT and NZMAT included observers from regional EMTs. A hybrid approach was applied for FEMAT’s reclassification visit, with the EMT secretariat from the WHO Regional Office for the Western Pacific participating online while WHO country office personnel and peer reviewers joined in person. This led to significant cost savings, reducing international travel for reclassifications.

Reclassification also allows for the field visit to take place during a real emergency deployment, local conditions permitting. This approach was tested in February 2023 during the Türkiye earthquake response, where JDR, along with EMTs from Italy and Spain, successfully underwent a reclassification field visit while their full EMT Type 2 was deployed. Following the field visit in February 2023, JDR completed the submission and online desk review of the EMT’s improvement dossier in November 2023. Peer reviewers for the online desk review were from the Italian EMT Type 2 from Regione Piemonte and the global and Western Pacific regional EMT secretariats. This approach, considered a pilot, was subsequently approved by the EMT SAG in October 2023 for future reclassifications (unpublished).

## OUTCOMES

Three EMTs in the Western Pacific Region were successfully reclassified in 2023: AUSMAT, JDR and NZMAT; FEMAT completed reclassification in March 2025. (*5*) All four teams completed the requirements for reclassification and demonstrated high levels of compliance with the EMT guiding principles and standards, as well as extensive improvements since their initial classifications. Through this process, EMTs continue their engagement with the EMT network and maintain strong ongoing commitments to quality care in emergencies.

During all of these reclassification exercises, EMTs demonstrated how learning through initial classification and deployment experiences led to improvements that benefit patients in emergencies. Reclassified EMTs presented strengthened capacities in both technical and operational areas. AUSMAT substantially enhanced their ability to deliver oxygen to hospitalized patients and expanded their rehabilitation capacity, building on lessons from the 2019 Samoa measles outbreak and COVID-19 responses. (*5*, *7*) All EMTs also showed improved logistics capacities, such as more robust tents, improved temperature controls, and expanded and more reliable systems for water, sanitation and hygiene. One reclassified EMT, while demonstrating redundant communications capabilities, continued to struggle with administrative hurdles to maintain satellite communications subscriptions. (*8*) Following the reclassification visits, all EMTs receive reports highlighting strengths and areas for improvement, which will serve as a point of reference in future 5-yearly reclassification exercises.

The process of reclassification helps to maintain strong engagement between the EMTs and the WHO secretariat and reinforces peer-to-peer support through mentoring, peer review and shared commitment to continuous improvement. Through reclassification, all four EMTs demonstrated continuous compliance with published EMT standards and received recommendations from reviewers on further opportunities for improvement.

## Discussion

The EMT classification and reclassification processes aim to ensure that populations affected by emergencies receive timely, high-quality and safe care, and that countries requesting and receiving international EMTs have predictability and confidence in the deploying teams. Through the EMT reclassification process, AUSMAT, FEMAT, JDR and NZMAT demonstrated strong commitments to upholding EMT principles and standards and reflected strong commitments to continuous improvement and innovation.

The experience of EMT reclassification in the Western Pacific Region demonstrates the value of continued quality assurance, peer review and commitments to continuous learning and improvement among EMTs. The reclassification process, which was designed to be “lighter” than the initial classification, can also be flexible in the sequencing of steps, as seen by JDR undergoing a field visit during deployment, and FEMAT piloting a hybrid verification team for their site visit with the virtual presence of the regional WHO EMT secretariat. The EMT SAG and secretariat aim to continue refining these processes to reduce costs and enhance flexibility while ensuring continued quality assurance. Additional research, reflection and publication by individual teams on their own reclassification processes would serve as valuable additions to the literature on EMTs and health emergency preparedness and response.

By regularly reviewing continued compliance with WHO-EMT standards, countries can enhance their capacities to provide emergency health care that aligns with local clinical protocols, integrates with local health systems and works towards greater interoperability between medical and public health capacities. (*4*-*6*, *9*, *10*)
